# Recent Advances of SnO_2_-Based Sensors for Detecting Volatile Organic Compounds

**DOI:** 10.3389/fchem.2020.00321

**Published:** 2020-05-05

**Authors:** Baoliang Li, Qu Zhou, Shudi Peng, Yiming Liao

**Affiliations:** ^1^College of Engineering and Technology, Southwest University, Chongqing, China; ^2^Chongqing Electric Power Research Institute, State Grid Chongqing Electric Power Company, Chongqing, China

**Keywords:** SnO_2_ based sensor, gas detection, VOCs, nanomaterials, improvement strategies

## Abstract

SnO_2_ based sensors has received extensive attention in the field of toxic gas detection due to their excellent performances with high sensitivity, fast response, long-term stability. Volatile organic compounds (VOCs), originate from industrial production, fuel burning, detergent, adhesives, and painting, are poisonous gases with significant effects on air quality and human health. This mini-review focuses on significant improvement of SnO_2_ based sensors in VOCs detection in recent years. In this review, the sensing mechanism of SnO_2_-based sensors detecting VOCs are discussed. Furthermore, the improvement strategies of the SnO_2_ sensor from the perspective of nanomaterials are presented. Finally, this paper summarizes the sensing performances of these SnO_2_ nanomaterial sensors in VOCs detection, and the future development prospect and challenges is proposed.

## Introduction

Volatile Organic Compounds (VOCs) are the most crucial cause of indoor air pollution and harm to human health, including a variety of toxic compounds and carcinogens (Shrubsole et al., 2019). For example, organic waste gases such as formaldehyde and polycyclic aromatic hydrocarbons have strong carcinogenicity, when the human body is in this environment for a long time, the possibility of carcinogenesis will greatly increase. There are also some organic waste gas shows a strong toxic effect, the human body in the excessive inhalation, will lead to coma or even death (Li G. et al., 2019). In addition, VOCs exhaust gas may also cause environmental problems such as acid rain, ozone layer damage, and atmospheric warming (Meng et al., 2018). Therefore, it is very essential to analyze the composition and concentration of VOCs in the air. Current methods for detecting VOCs include Gas Chromatography (GC), Gas Chromatography-mass spectrometry (GC-ms) and gas sensor detection (Vesely et al., 2003; Teixeira et al., 2004). GC determination of a single sample requires reference to known standards, and GC-ms requires a high time cost and complicated process. More importantly, both methods are offline and cannot detect the content and change of VOCs in real-time. In recent years, gas sensors has been widely noticed because of its fast detection speed, small volume, simple measurement and on-line monitoring.

Carbon materials and metal oxide semiconductor materials like SnO_2_, ZnO, WO_3_, and In_2_O_3_ have received scientific and technological importance and are widely used to detect VOCs gases (Luo et al., 2016; Lin et al., 2019; Zhao et al., 2019b). SnO_2_ gas sensor has been extensively studied for its applications in air quality detection, flammable and explosive gas detection, and environmental monitoring (Zhang Q. Y. et al., 2018; Zhou et al., 2018c). Nanomaterials have become the focus of the best sensing materials in recent years. Nanomaterials have many natural advantages such as large specific surface area, small size, and lightweight (Lu et al., 2018a; Zhou et al., 2018a). At present, there are many kinds of structures such as nanowires, nanofilaments, nanowires hollow spheres, nanofilaments flowers, and nanotubes (Mirzaei et al., 2016; Zhang Q. Y. et al., 2017). Different nanostructures and morphologies have different effects on the properties of materials. In order to change the nanostructure of a single material, there are other ways to improve the gas sensitivity of the sensor. This mini-review summaries the gas-sensing performances of SnO_2_ based sensor, which were influenced by the microstructure, doping, oxide composite and noble metal modification, toward toluene (C_6_H_5_CH_3_), formaldehyde (HCHO), and acetone (C_3_H_6_O).

## Sensing Mechanism of SnO_2_ Gas Sensor

SnO_2_ sensor is a surface-controlled gas sensor. The gas-sensing reaction can only cause changes in parameters such as surface conductivity of the semiconductor (Ducere et al., 2012; Korotcenkov and Cho, 2017). When exposed to air, oxygen molecules would be adsorbed on the surface of the SnO_2_ nanostructures and capture electrons from the conduction band of SnO_2_ to generate chemisorbed oxygen species [${\text{O}}_{2}^{-}$, O^−^, and O^2−^, depending on temperatures; (Shahabuddin et al., 2017; Zhou et al., 2019)]. The chemical adsorption process can be explained by the following reactions:

(1) $$\begin{array}{l} O_{2}\left(gas\right)↔O_{2}\left(ads\right) \end{array}$$

(2) $$\begin{array}{l} O_{2}\left(ads\right)+e^{-}↔{O_{2}}^{-}\left(ads\right)\left(T<150^{\circ }C\right) \end{array}$$

(3) $$\begin{array}{l} {O_{2}}^{-}\left(ads\right)+e^{-}↔2O^{-}\left(ads\right)\left(150^{\circ }C<T<400^{\circ }C\right) \end{array}$$

(4) $$\begin{array}{l} O^{-}\left(ads\right)+e^{-}↔O^{2-}\left(ads\right)\left(T>400^{\circ }C\right) \end{array}$$

When SnO_2_ sensor contacts with the measured gas, its resistance will change according to the oxidation or reduction characteristics of the gas. Toluene, formaldehyde and acetone tested in this paper are reductive gases. When SnO_2_ material surface comes into contact with a reducing gas, the reducing gas will react with oxygen anions to produce carbon dioxide and water, and the resulting electrons will return to the conduction band of the semiconductor. Therefore, this process will increase the carrier concentration on the surface of SnO_2_ material, resulting in a decrease in the resistance value. When finally restored to the air environment, the sensor returns to its original state (Lu et al., 2018b; Al-Hashem et al., 2019; Mahajan and Jagtap, 2019). The sensing mechanism of the SnO_2_ sensor reacting with these gases can be represented by the following path, where O^−^ is taken as an example (Lian et al., 2017; Zhu et al., 2019):

(5) $$\begin{array}{l} C_{6}H_{5}CH_{3}+18O^{-}\to 7CO_{2}+4H_{2}O+18e^{-} \end{array}$$

(6) $$\begin{array}{l} HCHO+2O^{-}\to CO_{2}+H_{2}O+2e^{-} \end{array}$$

(7) $$\begin{array}{l} C_{3}H_{6}O+8O^{-}\to 3CO_{2}+3H_{2}O+8e^{-} \end{array}$$

## Optimization of SnO_2_ Gas-sensing Materials

**Figure 1:**
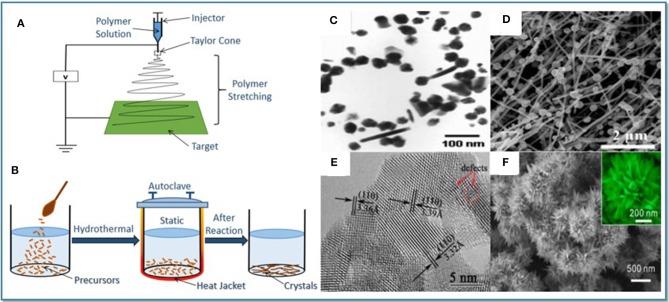
**(A)** A schematic of electrospinning method. **(B)** A schematic of hydrothermal method. **(C)** SnO_2_ nanoparticles. Reprinted with permission from Matussin et al. (2020). **(D)**SnO_2_/ZnO nanofibers. Reprinted with permission from Li H. et al. (2019). **(E)** SnO_2_ nanosheets. Reprinted with permission from Zhu et al. (2015). **(F)** SnO_2_ tapered layered nanostructures. Reprinted with permission from Li et al. (2017).

With the development of semiconductor gas-sensing materials, it has been the focus of research to enhance their gas-sensing properties for gas detection. The most common preparation methods of SnO_2_ sensing materials include electrostatic spinning and hydrothermal methods, as shown in Figures 1A,B. Different preparation methods will change the structure and morphology of SnO_2_ sensing materials and further enhance the gas sensitivity (Long et al., 2018; Zhang Y. J. et al., 2018; Zhou et al., 2018b). This section mainly reflects the changes in the gas-sensing properties of SnO_2_ nanomaterials from the aspects of structure and morphology design, ion doping, oxide composite and noble metal modification (Chen et al., 2013; Das and Jayaraman, 2014).

Different nanostructures and morphologies cause various effects on the properties of materials. In this respect, various morphologies from 0-D to 3-D with unique physical and chemical properties have been successfully synthesized. High dispersivity, ultra-small diameter 0-D SnO_2_ nanoparticles (Figure 1C) have highly effective surface areas and sufficient oxygen vacancies, which can improve the performance of nanoparticle based sensors (Matussin et al., 2020). 1-D SnO_2_ structure, such as nanofibers (Figure 1D), has excellent sensitivity and stability due to its large specific surface area, high porosity and good permeability (Li H. et al., 2019). Compared with low dimensional structure, 2-D structure possesses larger special surface area. In comparison to the 3-D structure, freestanding 2-D structures such as SnO_2_ nanosheets (Figure 1E) can provide better optimization including the modulation of the materials activity, surface polarization, and rich oxygen vacancies (Zhu et al., 2015). 3-D structures, such as microspheres, microflowers, and mesoporous structures, are assembled by diverse lower dimension fundamental blocks. Microstructured analyses suggest that the favorable gas sensitivity of SnO_2_ tapered layered nanostructures (Figure 1F) are mainly ascribed to the formation of more active surface defects and mismatches (Li et al., 2017).

Ion doping can change the cell parameters of the material, the number of suspensions on the surface of the material and the richness of defects, thereby enhance the gas sensitivity of the sensor (Korotcenkov and Cho, 2014). The Y-doped SnO_2_ three-dimensional flower-like nanostructure prepared by one-step hydrothermal method has a large number of rough nanoflakes, which increases the specific surface area and is more conducive to the adsorption and desorption of oxygen and formaldehyde gas. It is a highly sensitive formaldehyde detection material (Zhu et al., 2019). The doping of Ce ions into the SnO_2_ lattice results in the smaller size of nanoparticles and the formation of a porous structure. Therefore, Ce ions can provide more active sites for the adsorption and reaction of acetone (Lian et al., 2017).

The combination of two metal oxides can improve the gas sensitivity of semiconductor materials. The dispersion of functional components is the key factor to realize good gas sensitivities (Wei et al., 2020). Moreover, the heterostructure formed by SnO_2_ and another metal oxide can promote the transfer of carriers between materials and change the conductivity and energy band structure of composite materials (Gusain et al., 2019; Wei et al., 2019). In a recent research, a facile solvent EIOC has been demonstrated for the synthesis of novel hierarchical branched mesoporous TiO_2_-SnO_2_ semiconducting heterojunctions. The uniform distribution of SnO_2_ NCs in the pore walls of TiO_2_ forms numerous n-n heterojunctions which are extremely useful for surface catalytic reaction. Owing to the rational combination of a hierarchical mesoporous structure, a high crystallinity, and well-defined n-n heterojunctions, the SHMT-based gas sensor shows an excellent sensing performance with a fast response and recovery dynamics, ultralow limit of detection and a superior selectivity (Zhao et al., 2019a). The cactus-like WO_3_-SnO_2_ nanocomposite was prepared by one-step hydrothermal method by attaching many tiny SnO_2_ nanospheres to large WO_3_ nanospheres, which provided many active sites for the acetone molecule and provided heterojunctions between WO_3_ and SnO_2_. The synergistic effect between them improves the sensing performance of the composite nanomaterial to acetone gas (Zhu et al., 2018).

Precious metal modification usually uses Au, Ag, Pt, and Pd or their oxides to improve the sensitivity and response speed of gas sensing materials and reduce the working temperature. Ag modified SnO_2_ nanoparticles prepared by hydrothermal *in situ* reduction improved the sensor's ability to detect formaldehyde. This is due to the charge transfer between Ag and SnO_2_, which increases the absorption band on the composite by 20 nm, thus improving the gas sensitivity (Liu et al., 2019). When the acetone is detected by Ag/SnO_2_ porous tubular nanostructures prepared by electrospinning, the sensor resistance changed rapidly and significantly. On the one hand, the p-n hybrid interface of p-type Ag_2_O and n-type SnO_2_ causes the energy band of the depletion layer to bend, increasing the initial resistance. On the other hand, the hollow nanostructure promotes the adsorption and electron transfer of acetone, which makes the resistance change rapidly (Xu et al., 2017). The bimetal PdAu modified SnO_2_ nanosheet showed excellent selectivity and responsiveness to low concentrations of acetone, which is due to the chemical sensitization of Au, electronic sensitization of Pd and synergism of PdAu bimetal nanosheet (Li G. et al., 2019).

## Sensing Performance of VOCs Based On SnO_2_ Nanomaterials

For VOCs, this review mainly introduces toluene, formaldehyde and acetone. This section summarizes the gas-sensitive characteristics of SnO_2_-based nanomaterials for the above gases, as shown in Table 1. In the detection of common VOCs, the lower detection limit, response value and detection temperature of SnO_2_ based nanomaterials are different.

**Table 1:** Comparison of SnO_2_ based nanomaterials for VOCs detection.

**VOCs**	**Material**	**Synthesis method**	**Detection limit**	**Response**	**Temperature (^**°**^C)**	**References**
Toluene	Pd-doped SnO_2_ hollow spheres	One-pot hydrothermal method	100 ppb	52.9 (20 ppm)	230	Zhang K. et al., 2017
	Micro-/mesoporous SnO_2_ spheres	Solvothermal method	10 m	20.2 (50 ppm)	400	Hermawan et al., 2019
	Pd-loaded SnO_2_ cubic nanocages	Multi-step route	100 ppb	41.4 (20 ppm)	250	Qiao et al., 2017
	Pd/SnO_2_ nanofibers	electrospinning and carbonization	0.5 ppm	24.6 (100 ppm)	250	Xie et al., 2018
Formaldehyde	Ag-SnO_2_ composites	Hydrothermal and *in situ* reduction method	10 ppm	14.4 (10 ppm)	125	Liu et al., 2019
	Ag doped Zn_2_SnO_4_/SnO_2_ hollow nanospheres	Hydrothermal method	5 ppm	62.2 (50 ppm)	140	Zhang et al., 2019
	Ni doping of SnO_2_ nanoparticles	Hydrothermal method	1 ppm	130 (100 ppm)	200	Hu et al., 2018
	Y-doped SnO_2_ flower-shaped nanostructures	Hydrothermal method	1 ppm	18 (50 ppm)	180	Zhu et al., 2019
	NiO-SnO_2_ heterojunction microflowers	Hydrothermal method	1 ppm	39.2 (100 ppm)	100	Meng et al., 2018
	Cedar-like SnO_2_ hierarchical micro-nanostructures	Low-temperature hydrothermal method	5 ppm	13.3 (100 ppm)	200	Yu et al., 2017
	GO/SnO_2_ nanocomposites	Electrospinning and calcination procedure	500 ppb	32 (100 ppm)	120	Wang et al., 2017
	SnO/SnO_2_ nano-flowers	Hydrothermal method	8.15 ppb	80.9 (50 ppm)	120	Li N. et al., 2019
	Cd-doped SnO_2_ nanofibers	Hydrothermal method	1 ppm	51.11 (100 ppm)	160	Zhao et al., 2020
Acetone	Ca^2+^/Au co-doped SnO_2_ nanofibers	Electrospinning and calcination procedure	10 ppm	62 (100 ppm)	180	Jiang et al., 2017
	La_2_O_3_-doped SnO_2_ nanoparticulate thick films	Flame-spray-made	100 ppb	3,626 (400 ppm)	350	Tammanoon et al., 2018
	Ce-doped SnO_2_ nanoparticles	Hydrothermal method	10 ppm	50.5 (50 ppm)	270	Lian et al., 2017
	Ag-decorated SnO_2_ hollow nanofibers	Electrospinning method	5 ppm	117 (200 ppm)	160	Xu et al., 2017
	Au@WO_3_-SnO_2_ corrugated nanofibers	Hydrothermal treatment process	200 ppb	79.6 (0.5 ppm)	150	Shao et al., 2019
	PdAu decorated SnO_2_ nanosheets	*In situ* reduction method	45 ppb	109 (50 ppm)	250	Li G. et al., 2019
	Cactus-like WO_3_-SnO_2_ nanocomposite	Hydrothermal method		26 (600 ppm)	360	Zhu et al., 2018

Toluene, a colorless volatile liquid, is one of the most widely used aromatic hydrocarbons and is considered as a biomarker of cancer. Occupational Safety and Health Administration (OSHA) stipulates that the permissible exposure limit for toluene is 100 ppm for 8 h (Sui et al., 2017). The Pd-doped SnO_2_ hollow spheres prepared by hydrothermal method measured a response value of 52.9 for toluene at 20 ppm and a lower temperature of 230°C (Zhang K. et al., 2017). The Pd-loaded SnO_2_ cubic nanocages are also an ideal choice for toluene detection, with a minimum detection concentration of 100 ppb, a response to 20 ppm of toluene of 41.4, and an optimal reaction temperature of 250°C (Qiao et al., 2017). Formaldehyde is a colorless and pungent gas. Due to the toxicity of formaldehyde, OSHA has established the Threshold Limit Value (TLV) as a concentration of 0.75 ppm for 8 h. The SnO/SnO_2_ nano-flowers prepared by hydrothermal method have a minimum detection concentration of 8.15 for formaldehyde, an optimal response temperature of 120°C, and a response value of 80.9 at 50 ppm. It is an ideal material for formaldehyde detection (Li N. et al., 2019). The hydrothermal Ni doping of SnO_2_ nanoparticles also had a good response value of 130–100 ppm of formaldehyde at 200°C (Hu et al., 2018). In addition, the Ag-doped Zn_2_SnO_4_/SnO_2_ hollow nanospheres responded to 50 ppm of formaldehyde with a value of 62.2 and a lower detection temperature of 140°C (Zhang et al., 2019). Acetone is a colorless and irritant liquid. Long term exposure to acetone can stimulate human sensory organs and lead to inflammation. Therefore, the quantitative detection of acetone is of great significance (Cheng et al., 2015; Lian et al., 2017). PdAu decorated SnO_2_ nanosheets sensor was able to detect acetone at 45 ppb and to respond to acetone at 50 ppm to 109 (Li G. et al., 2019). The detection limit of Au @ WO_3_-SnO_2_ corrugated nanofibers prepared by hydrothermal treatment was 200 ppb acetone, and the best response to 0.5 ppm acetone at 150°C was 79.6. The Au@WO_3_-SnO_2_ corrugated nanofibers is an ideal low concentration acetone gas sensor with low detection limit and high response (Shao et al., 2019). La_2_O_3_-doped SnO_2_ nanoparticle thick films has an amazing response value of 3,626 −400 ppm at 350°C, which is suitable for the detection of high concentration acetone (Tammanoon et al., 2018).

## Conclusion and Perspective

This review discusses the performance improvements of SnO_2_-based nanomaterials and the comparison of gas sensitivity in VOCs in recent years. SnO_2_-based nanostructures provide a larger specific surface area and more active sites, which is conducive to VOCs adsorption. Ion doping can reduce the size of nanomaterials and make the surface of the material rougher, thereby increasing the specific surface area. Metal oxide composite can not only achieve functional dispersion, but also form heterojunctions to promote the movement of charge carriers. Precious metals have excellent catalytic activity for SnO_2_ nanomaterials. These optimization methods make SnO_2_-based gas sensors operate at lower temperatures, higher sensitivity, and better stability. Despite great progress has been made in the application of SnO_2_ nanomaterials, there is still much room for further development. First of all, cross sensitivity is a huge challenge for the preparation of high-performance sensors. In the future, SnO_2_ gas sensor will be able to detect a single gas in the mixture. Secondly, most of the SnO_2_ sensors currently used work at high temperature, which limits their wide application in detecting VOCs at room temperature. In addition, long-term stability is also one of the research hotspots of SnO_2_ sensors in the future. Due to the influence of external environment and other factors, the stability of the sensor can not be guaranteed. Therefore, it is of great significance to develop more stable gas sensors. It has become a research hotspot to optimize the existing gas sensing materials by chemical modification and develop new gas sensing materials such as composite and hybrid semiconductor materials and polymer gas sensing materials. In addition, new sensors, such as optical waveguide gas sensor, quartz resonant gas sensor and microbial gas sensor, developed with advanced processing technology and microstructure, can make the sensor more stable, and versatility. Finally, we hope our work will be helpful for the further exploration of metal oxide nanomaterials in the detection of VOCs.

## Author Contributions

All authors listed have made a substantial, direct and intellectual contribution to the work, and approved it for publication.

## Conflict of Interest

The authors declare that the research was conducted in the absence of any commercial or financial relationships that could be construed as a potential conflict of interest.
